# Waldenström Macroglobulinemia and Cerebral Venous Thrombosis: From Diagnosis to Complication

**DOI:** 10.1155/2019/9581605

**Published:** 2019-07-14

**Authors:** Paulo Zoé Costa, Pedro Chorão, Andreia Póvoa, Pedro Vieira, Heidy Cabrera, Orlando Mendes, Céu Evangelista

**Affiliations:** ^1^Department of Internal Medicine, Unidade Local de Saúde da Guarda E.P.E., Guarda 6301-857, Portugal; ^2^Department of Clinical Hematology, Centro Hospitalar São João E.P.E., Porto 4200-319, Portugal; ^3^Department of Internal Medicine, Centro Hospitalar da Cova da Beira E.P.E., Covilhã 6200-251, Portugal

## Abstract

Waldenström macroglobulinemia (WM) is a type of non-Hodgkin lymphoma in which cancer cells produce large amounts of an abnormal protein that can cause hyperviscosity syndrome (HVS). A 43-year-old woman with WM, who developed seizures, had a head computed tomography scan that showed signs of cerebral venous thrombosis (CVT). Nevertheless, the value of immunoglobulin M was lower than 50 g/L, and evaluation of serum viscosity was not performed. Moreover, there was no history of bleeding, and the eye funduscopy was normal. These findings lead to think of causes of CVT other than HVS in a patient with WM.

## 1. Introduction

Waldenström macroglobulinemia (WM) is a B-cell lymphoproliferative disorder characterized by the co-occurrence of two main components: a bone marrow infiltration by lymphoplasmacytic lymphoma (LPL) and the presence of immunoglobulin M (IgM) paraprotein. The most common symptom is fatigue related to a normocytic anemia. Less commonly, several signs and symptoms are related to the excess IgM causing a condition known as hyperviscosity syndrome (HVS). Also, the accumulation of lymphoplasmacytic cells in different tissues can cause hepatosplenomegaly and lymphadenopathy [1, 2].

HVS is a clinical feature present in up to 30% of patients with WM and results from the several mechanisms related to physicochemical and immunologic properties of the monoclonal IgM. The symptoms include skin and mucosal bleedings, visual disturbance secondary to retinopathy, neurological symptoms (headache, vertigo, dizziness, nystagmus, deafness, and ataxia), and, in rare cases, congestive cardiac failure [3]. Cerebral venous thrombosis (CVT) can also appear as clinical manifestation in cancer patients, particularly in patients with hematologic malignancies [4].

There is no definitive etiology for WM, but in some cases, it is associated with infection by hepatitis C virus. The disease may be preceded by IgM monoclonal gammopathy of undetermined significance. Identification of an M-protein is usually carried out by serum protein electrophoresis (SPEP) and must be confirmed by the more sensitive method of immunofixation. A bone marrow aspirate and trephine biopsy assessments are required for definitive diagnosis.

Although neither specific nor required, more than 90% of LPLs have MYD88 L265P mutation, which can aid in the diagnosis [1].

This paper aims to discuss the cause and relationship of CVT in patients with WM due to HVS and hypercoagulable state.

## 2. Case Presentation

A 43-year-old woman with a history of fatigue was referred to our outpatient department by her general practitioner. Her past medical history was unremarkable. She smoked 15 cigarettes daily for 20 years (15 pack‐year) and took gestodene/ethinylestradiol for birth control (above 15 years). Her physical examination was normal. The laboratory study (Table 1) revealed a normocytic normochromic anemia, an M-spike in the gamma region of SPEP, elevation of serum IgM and serum kappa free light chain, and elevation of serum beta-2 microglobulin.

Bone marrow biopsy confirmed an infiltration by lymphoplasmacytic B cells, with immunohistochemistry compatible with LPL. Molecular testing confirmed the presence of MYD88 L265P mutation. She was referred to Hematology to initiate chemotherapy with dexamethasone, rituximab, and cyclophosphamide (DRC; each cycle consists of a 21-day course of dexamethasone 20 mg intravenously followed by rituximab 375 mg/m^2^ intravenously on day 1 and cyclophosphamide 100 mg/m^2^ orally bid on days 1 to 5).

After 5 cycles (15 weeks) of DRC, she was admitted to the Emergency Department for a first episode of convulsive seizure. She complained of headaches, nausea, and vomiting since the week before. During physical examination, the patient was tachycardic on auscultation and had unilateral dysmetria on the finger-nose test, with no visual impairment and normal funduscopy. Her chest X-ray was normal, and her head computed tomography scan showed signs of CVT in the superior sagittal sinus, torcula, right transverse sinus, and right sigmoid sinus. The patient was admitted for further evaluation. The laboratory findings at admission are shown in Table 2.

Holter monitoring, transthoracic echocardiogram, and Doppler ultrasound of the neck blood vessels were performed and identified no alterations. Other prothrombotic conditions, such as infections and autoimmune diseases, were ruled out, and tests for thrombophilia (e.g., lupus anticoagulant, anti-cardiolipin antibody, anti-*β*2 glycoprotein 1 antibody, activated protein C resistance, fibrinogen tests, factor V Leiden, prothrombin mutation, and basal homocysteine levels) were negative.

Serum viscosity is not available at our institution. The IgM value was 16.40 g/L, and the patient did not require plasma exchange.

Symptomatic treatment, plenty of fluids, and anticoagulation with enoxaparin were started.

## 3. Discussion

The cause of CVT in this patient is multifactorial: first, she took combined oral contraceptive pill gestodene/ethinylestradiol for more than 15 years, which increased the risk of venous thromboembolism. Second, she was smoker. Moreover, hematologic malignancies and chemotherapy can increase the risk of thrombosis and CVT [4]. Experimental studies show that glucocorticoids increase levels of clotting factors and fibrinogen by damaging the endothelium, thereby predisposing it to clotting [5]. This patient has received 5 cycles of chemotherapy in which dexamethasone 20 mg was given intravenously as part of the combination.

It is interesting to discuss if HVS could have been the cause of CVT in this case because HVS is associated most commonly with WM. IgM is a star-shaped pentamer with a molecular size of 925 kDa, while albumin is 65 kDa. Thus, this molecule, which is 80% intravascular, exerts profound effects on blood flow and cells, especially when present in high concentrations as in WM patients [3]. The IgM paraprotein also interacts with red cells, platelets, coagulation factors, and adhesive molecules and thereby can promote formation of thrombi [6].

Symptoms usually occur when the monoclonal IgM concentration exceeds 50 g/L or when plasma viscosity (PV) is greater than 4.0 centipoises (cp). Nevertheless, there is individual variability, such as the condition of blood vessels (e.g., stiffness), which may play a role and explain why some patients show no evidence of hyperviscosity at 10 cp of PV or, on the opposite, why some patients develop symptoms of HVS with lower levels of IgM [2, 7].

Evaluation of serum viscosity was not performed in this patient because the laboratory in our hospital does not provide such measurement and the value of IgM was lower than 50 g/L. Moreover, there was no history of bleeding, and the eye funduscopy was normal. The clinical features were only neurological symptoms, such as convulsive seizure.

On the other hand, other causes were ruled out. Also, she presented with nausea and vomiting for about one week, which could have worsen the situation owing to dehydration, increasing blood viscosity [8]. In addition, serum viscosity is not always proportional to the concentration of IgM, as mentioned above. And finally, the serum concentration of the IgM paraprotein decreased significantly from 45.7 g/L to 16.4 g/L after 5 DRC treatment courses, which makes serum viscosity as a provocative trigger over time less likely (Table 2).

## 4. Conclusion

The cause of CVT in this patient was probably caused by her hypercoagulable state due to the reasons explained above, and not HVS. HVS is a complication that should be considered in patients with WM, but in this case, it had a small role in development of CVT and it was important to investigate other causes.

## Figures and Tables

**Table 1:** Laboratory investigations at diagnosis of Waldenström macroglobulinemia.

Leucocytes	4.85 g/L	5.0–10.0
Haemoglobin	11.1 g/dL	12.0–16.0
Hematocrit	33.6%	36–46
MCV	85.7 fL	80–100
RDW	15.2%	11.9–14.5
Thrombocytes	280 × 10^3^/*μ*L	140–400 × 10^3^
Urea	32 mg/dL	15–40
Creatinine	0.87 mg/dL	0.57–1.11
Uric acid	2.76 mg/dL	2.6–6.0
IgG	7.19 g/L	7.51–15.6
IgA	3.77 g/L	0.82–4.53
IgM	45.70 g/L	0.40–2.74
Kappa free light chain	30.10 g/L	6.29–13.50
Lambda free light chain	<0.005 g/L	0.006–0.026
Ratio free kappa/lambda	15.4	
Beta-2 microglobulin	0.32 mg/dL	0.11–0.24

Urine 24 h		
Kappa light chain	0.743 g/L	<0.185
Lambda light chain	<0.05 g/L	<0.050
Kappa free light chain	0.248 g/L	0.039–1.51
Lambda free light chain	<0.011 g/L	0.081–1.01

MCV, mean corpuscular volume; RDW, red cell distribution width; Ig, immunoglobulin.

**Table 2:** Laboratory investigations at diagnosis of cerebral venous thrombosis.

Leucocytes	3.87 g/L	5.0–10.0
Haemoglobin	11.0 g/dL	12.0–16.0
Hematocrit	32.8%	36–46
MCV	86.8 fL	80–100
RDW	14.6%	11.9–14.5
Thrombocytes	180 × 10^3^/*μ*L	140–400 × 10^3^
Urea	14 mg/dL	15–40
Creatinine	0.77 mg/dL	0.57–1.11
Uric acid	2.76 mg/dL	2.6–6.0
IgG	1.72 g/L	7.51–15.6
IgA	0.641 g/L	0.82–4.53
IgM	16.40 g/L	0.40–2.74
Kappa free light chain	0.019 g/L	0.003–0.019
Lambda free light chain	<0.005 g/L	0.006–0.026
Ratio free kappa/lambda	3.8
Beta-2 microglobulin	0.24 mg/dL	0.11–0.24

Urine 24 h		
Kappa light chain	0.0441 g/L	<0.0185
Lambda light chain	<0.05 g/L	<0.05
Kappa free light chain	0.032 g/L	0.039–1.51
Lambda free light chain	<0.005 g/L	0.081–1.01

MCV, mean corpuscular volume; RDW, red cell distribution width; Ig, immunoglobulin.
